# In Vitro Antifungal Activity of Plant Extracts on Pathogenic Fungi of Blueberry (*Vaccinium* sp.)

**DOI:** 10.3390/plants10050852

**Published:** 2021-04-23

**Authors:** Abraham Hernández-Ceja, Pedro Damián Loeza-Lara, Francisco Javier Espinosa-García, Yolanda M. García-Rodríguez, José Roberto Medina-Medrano, Germán Fernando Gutiérrez-Hernández, Luis Fernando Ceja-Torres

**Affiliations:** 1 Instituto Politécnico Nacional, Centro Interdisciplinario de Investigación para el Desarrollo Integral Regional Unidad Michoacán, Jiquilpan, MI 59510, Mexico; Abm_hernandez@hotmail.com; 2 Licenciatura en Genómica Alimentaria, Universidad de La Ciénega del Estado de Michoacán de Ocampo, Sahuayo, MI 59103, Mexico; pdloeza@ucienegam.edu.mx; 3 Instituto de Investigaciones en Ecosistemas y Sustentabilidad, Universidad Nacional Autónoma de México, Morelia, MI 58190, Mexico; espinosa@cieco.unam.mx (F.J.E.-G.); ygarcia@cieco.unam.mx (Y.M.G.-R.); 4 CONACYT–Centro Interdisciplinario de Investigación Para el Desarrollo Integral Regional Unidad Michoacán, Instituto Politécnico Nacional, Jiquilpan, MI 59510, Mexico; jrmedina@conacyt.mx; 5 Instituto Politécnico Nacional, Unidad Profesional Interdisciplinaria de Biotecnología, Ticomán, CDMX 07340, Mexico; gfgutierrez@ipn.mx

**Keywords:** thin layer chromatography, column chromatography, antifungal extracts, gas chromatography-mass spectrometry, antifungal compounds

## Abstract

Three pathogenic fungi of blueberry (*Vaccinium* spp.) responsible for dieback disease, identified as *Pestalotiopsis clavispora*, *Colletotrichum gloeosporioides* and *Lasiodiplodia pseudotheobromae*, were isolated in the northwestern region of the state of Michoacán, Mexico. The mycelial growth in vitro of these fungi was inhibited by extracts from *Lantana hirta*, *Argemone ochroleuca* and *Adenophyllum porophyllum*, medicinal plants collected in Sahuayo, Michoacán, Mexico. The extracts showed different degrees of inhibition; the most effective were: M5L extract from *L. hirta* and M6LFr extract from *A. ochroleuca*, both of which inhibited 100% of the mycelial growth of *P. clavispora* and *C. gloeosporioides*; and M4LS extract from *A. porophyllum*, which inhibited 100% of the mycelial growth of the three pathogens. The extracts were fractionated by thin layer and column chromatography, and the most active fractions were analyzed by gas chromatography-mass spectrometry. The major compounds identified in *L. hirta* extract were Phytol and α-Sitosterol. The compounds identified in *A. ochroleuca* were Toluene and Benzene, 1,3-bis(3-phenoxyphenoxy)-. In *A. porophyllum*, the compound identified was Hexanedioic acid, bis(2-ethylhexyl) ester. These results show the potential of *L. hirta*, *A. ochroleuca* and *A. porophyllum* as a source of antifungal compounds.

## 1. Introduction

The main producers of blueberry are the United States, Canada and Poland; Mexico is ranked 18th. In this country, the major producing states are Michoacán and Jalisco, with 37,090 t produced in 2019 [1]. In the northwestern region of the state of Michoacán, Mexico, the production of blueberry is affected by various factors; among the most important are diseases caused by *Pestalotiopsis* sp.; *Colletotrichum* sp.; and other fungi belonging to the family Botryosphaeriaceae, such as *Lasiodiplodia theobromae*, *Neofusicoccum ribis* and *Botryosphaeria* sp. [2,3,4]. These fungi have the ability to affect fruits and stems, causing the disease known as dieback [5,6], which is responsible for significant losses in the production of blueberries and the increase in production costs related to fungicides. The continuous and indiscriminate use of fungicides causes adverse effects to human health and the environment [7], which, together with the increasing demand for blueberries, current regulations on chemical pesticides and the emergence of resistant pathogens, justifies the search for new active molecules that can be used as control agents against phytopathogenic fungi [7,8] and that can be included in programs associated with the integrated management of pests.

Secondary metabolites with fungicidal properties have biological activity against phytopathogenic fungi and are, therefore, a viable control option. They are biodegradable to non-toxic products and could be a sustainable tool as bio-pesticides in integrated pest management programs [9,10,11]. The main source of these metabolites can be found in medicinal plants, of which more than 200 species with this activity have been studied and reported in Mexico [12]. The state of Michoacán, Mexico, has an enormous wealth of plant species and important cultural traditions on the use of medicinal plants, such as “Siete Colores”, “Chicalote” and “Árnica de Cerro”, which are widely used as analgesics, and antidiarrheal, anti-inflammatory and antimicrobial remedies [13,14,15]. To our knowledge, no studies have been conducted to identify the bioactive secondary metabolites of these species or to evaluate their antifungal activity against the major pathogenic fungi of blueberries grown in the northwestern region of the state of Michoacán, Mexico.

Thus, the objectives of this study were to identify the main fungi responsible for causing dieback disease in blueberries; to evaluate the in vitro antifungal activity of extracts from *Lantana hirta*, *Argemone ochroleuca* and *Adenophyllum porophyllum* against fungi associated with this disease; and to identify the bioactive secondary metabolites present in these plant species.

## 2. Material and Methods

### 2.1. Isolation of Pathogenic Fungi of Blueberry

Diseased plants with symptoms of wilt, blight, canker and dieback were collected from plots in the following four municipalities that produce blueberries in the northwest of the state of Michoacán, Mexico: Emiliano Zapata, Los Palillos, La Magdalena and San Gregorio. The fungi were isolated according to the procedure previously published [16]. For this, fragments of diseased tissue of approximately 10 × 10 mm were obtained from the diseased plants collected; disinfested with 3% (*v/v*) sodium hypochlorite for 2 min, rinsed three times with sterile distilled water; and dried on sterile paper napkins for 20 min, in a laminar flow cabinet (CHC Biolus^®^, Daejeon, Korea). Five fragments of sick tissue were transferred per Petri dishes containing potato-dextrose-agar culture medium (PDA, Bioxon^®^, Estado de México, Mexico) and incubated at 28 °C for 4–7 days, until fungal growth. The fungal isolates were purified using the hyphal tip technique, as follows: the colonies were transferred to water-agar culture medium (20%) (Bioxon^®^), where they were left to grow for 3 days at 28 °C; hyphal tips were then removed and transferred individually to PDA medium. The fungi grown on PDA were kept on sterile filter paper (2 cm^2^) for 15 days. Finally, they were placed in a sterile container and left in refrigeration at 5 °C until further use.

### 2.2. Molecular Identification of Fungi

The molecular identification of the fungi was carried out by polymerase chain reaction (PCR) using oligonucleotides specific for the ITS regions of rDNA. The extraction and quantification of DNA was performed according to the method of Raeder and Broda [17]. The fungal DNA was purified using The Wizard^®^ Genomic DNA Purification Kit (A1120, Promega Corp., Madison, WI, USA). The PCR was performed using the primers ITS1-Fwd (5′-TCCGTAGGTGAACCTGCGG-3′) and ITS4-REV (5′-TCCTCCGCTTATTATTGATATGC-3′). PCR reactions were performed in a final volume of 25 µL using the GoTaq^®^ Green Master Mix kit (M7122, Promega Corp. Madison, WI, USA) 1 X; 5 µM of the primer ITS1-Fwd primer; 5 µM of ITS4-Rev; and 25 ng of genomic DNA. Amplification was performed in a C1000 thermal cycler (Bio-Rad^®^, Germany) under the following conditions: initial denaturation at 94 °C for 5 min, followed by 35 cycles of denaturation at 95 °C for 30 s, 58 °C for 30 s, 72 °C for 1 min and a final extension at 72 °C for 7 min. The PCR products were separated by agarose gel electrophoresis (2%) at 80 V for 40 min, and visualized using SYBR Gold^®^ (Invitrogen, Carlsbad, CA, USA). DNA fragments of 700 bp were selected for purification with ExoSAP-IT^®^ (N/P 78200, USB Affymetrix, Inc., Cleveland, OH, USA). The purified fragments were sequenced using primer ITS1-Forward (5′-TCCGTAGGTGAACCTGCGG-3′) and ITS4-Rev (5′-TCCTCCGCTTATTATTGATATGC-3′) with the ABI PRISM BigDye^®^ Terminator sequencing kit v3.1 (P/N 4336917, Applied Biosystems, Foster City, CA, USA). Fragment analysis was conducted in a Genetic Analyzer 3130 sequencer (Applied Biosystems^®^, HITACHI Tokyo, Japan). The sequences were assembled using SeqMan software 8 (LaserGene) (DNASTAR^®^, Madison, WI, USA) and analyzed using the GenBank database (www.ncbi.nlm.gov accessed on 8 May 2014).

### 2.3. Pathogenicity Tests

Pathogenicity tests were performed on months-old blueberry plants (cv. Biloxi) obtained from Centro Regional Universitario Centro Occidente (CRUCO) of Chapingo Autonomous University, Campus Morelia, Michoacán. Each plant was transplanted into plastic pots (8 L) with a soil (80%) and chicken manure vermicompost-based substrate (20%). Pure four days-old fungal cultures were used for inoculation. The experimental design consisted of randomized blocks with four treatments (three fungi and a single control with PDA only) and five repetitions. Three stems of each plant (experimental unit) were inoculated through a 1 cm incision on which a disc (5 mm in diameter) with PDA and mycelium of the corresponding strain was placed; parafilm was used to keep the disc firmly on the stem. The plants were inspected daily until the onset of symptoms.

### 2.4. Collection and Identification of Plants

The collection of Siete Colores, Chicalote and Árnica de Cerro was done during the flowering stage (July and August 2013) in Sahuayo, Michoacán, Mexico, located northwest of the state (20°03′ N and 102°44′ W and 1530 masl). The collected plants were identified by MSc Ignacio Garcia Ruiz as *Lantana hirta* (001-2013), *Argemone ochroleuca* (002-2013) and *Adenophyllum porophyllum* (003-2013); they are registered in the herbarium CIMI of CIIDIR-IPN Unidad Michoacán.

### 2.5. Obtaining Crude Extracts and Fractions

The plants were sectioned into roots, stems, leaves, flowers and fruits, which were disinfected with 1% sodium hypochlorite, rinsed with distilled water and dried with absorbent paper [18]. The crude extracts were obtained from the following plant organs: leaf (L) and flower (Fl) of *L. hirta*; leaf-fruit (LFr) and root (R) of *A. ochroleuca*; and leaf-stem (LS) and leaf of *A. porophyllum*. These organs were dehydrated in an oven (CRAFT^®^, Ciudad de México, Mexico) at 50 °C for three days; they were then left at room temperature (25 °C ± 2.0) for five days [19] and ground using a conventional blender. The extracts were obtained by maceration [20]; 15 g of the dry and ground material were mixed with 100 mL of absolute ethanol (EtOH, J.T. Baker^®^) or 100 mL of ethyl acetate (AcOEt, J.T. Baker^®^) to obtain each extract. Each mixture was placed in separate 200 mL beakers, which were kept in the dark and left to stand for 120 h at room temperature (25 °C ± 2.0). After this time, the extracts were filtered (Whatman^®^ filter paper no. 5) and dried at 50 °C in a rotary evaporator (Brinkmann/Büchi^®^). They were then weighed and all the extracts were re-dissolved in EtOH, adjusted to a concentration of 100 mg/mL and stored at 4 °C until further use.

The crude extracts with the highest antifungal activity were fractionated by thin layer chromatography (TLC) and column chromatography (CC). Thin layer chromatography was carried out using a mixture of hexane/ethyl acetate/methanol 80:20:1 *v/v* (J.T. Baker^®^), on aluminum plates precoated with silica gel 60 F254 (3.5 cm × 9 cm; Whatman^®^). After chromatography, the plates were left to dry outdoors and exposed to UV light (SPECTROLINE^®^, Spectronics Corporation, Westbury, New York, NY, USA). Finally, the plates were revealed with 10% sulfuric acid (Fermont^®^, Monterrey, NL, Mexico) to observe the bands [21].

Column chromatography was carried out using a glass column (50 cm in length) packed with 120 g of silica gel (Sigma^®^, St. Louis, MO, USA). The extract was eluted with a mixture of hexane-ethyl acetate-methanol 80:20:1 (*v/v*). The collected fractions were analyzed by TLC to verify their successful separation and each fraction was concentrated with nitrogen (N_2_) to 1 mL and stored at 5 °C.

### 2.6. Antifungal Activity Assays

A total of 12 extracts were obtained. Each extract was assayed with 0.5, 1, 2 and 5 mg/mL. The effect of the crude extracts on the mycelial growth of the fungi isolated from blueberry was evaluated according to the method proposed by Sánchez-Pérez et al. [22]. Each concentration was added to Petri dishes with PDA culture medium; 30 min after to allow the evaporation of EtOH and the diffusion of the extract, a disc of 5 mm in diameter with PDA and mycelium of the corresponding strain was placed in the center of the dish, which was then incubated at 28 °C. In addition, we included an absolute control (PDA + fungus), a negative control (PDA + fungus + EtOH) and a positive control (PDA + fungus + Thiabendazole 5 mg/mL, TECTO 60^®^ wettable powder, 600 g IA kg^−1^). All bioassays were performed in triplicate. The effect of extracts and fractions was determined by measuring the diameter of the colonies with a digital vernier (Fisher Scientific, Waltham, MA, USA), and expressed as the percentage inhibition of mycelial growth compared to the negative control, according to the formula: % inhibition = average mycelial growth of the control-average mycelial growth of the treatment/average mycelial growth of the control × 100 [23].

### 2.7. Identification of Bioactive Secondary Metabolites

The fractions with the strongest antifungal effect were analyzed by gas chromatography-mass spectrometry (GC-MS) using the fragrance method [24]. From each sample, 1 µL was analyzed with an Agilent 6890 (Agilent Technologies, Santa Clara, CA, USA) Gas Chromatography equipment with an HP-5MS (5% Phenyl 95% dimethylpolysiloxane) capillary column (30 m × 0.25 mm with 0.25 µm film thickness), which was coupled to an Agilent 5973N selective mass detector. Helium was used as a carrier gas at 7.67 psi with a 1.0 mL min^−1^ constant flow. The front inlet was maintained at 280 °C in a split ratio of 50:1. The initial oven temperature was set at 50 °C, which was increased to 280 °C at a rate of 5 °C min^−1^ and then held for 1 min at this constant temperature, subsequently increased to 380 °C at a rate of 25 °C min^−1^ and finally held at the temperature of 380 °C for 3 min. The mass spectrometer was operated in electrical ionization mode (EI), with a flow of 1 mL min^−1^, 70 eV ionization voltage, the interface temperature at 300 °C and a scan range of 50–500 m/z. The compounds were identified by comparing the mass spectra of each constituent with those stored in the NIST02.L database. We only accepted peaks with a degree of purity of one, and the spectra were identified using a concordance threshold of 90%. The relative abundance of each compound was estimated by dividing its peak area by the sum of the areas of all peaks detected in the sample (total peak area).

### 2.8. Statistical Analysis

All treatments were arranged in a completely randomized design. The data of percent inhibition of mycelial growth were transformed with the arcsine: √ percent inhibition/100 [25]. Analysis of variance (*p* ≤ 0.05) and Tukey’s test (*p* ≤ 0.05) were performed using the SAS statistical package (V. 9.0., SAS Inst. Inc., Cary, NC, USA).

## 3. Results

### 3.1. Identification of Pathogenic Fungi of Blueberry and Pathogenicity Tests

Three pathogens of blueberry were identified at the molecular level: *Pestalotiopsis clavispora*, *Colletotrichum gloeosporioides* and *Lasiodiplodia pseudotheobromae*. The three fungi inoculated into blueberry plants caused dark-colored lesions on the stems, loosening of the epidermis and brown coloration of the leaf-tips (cancers and dieback disease). The fungi were reisolated and identified from the lesions they caused to the plants, fulfilling Koch’s postulates.

### 3.2. Antifungal Effect In Vitro of Crude Extracts on the Mycelial Growth of Blueberry Pathogens

**Figure 1:**
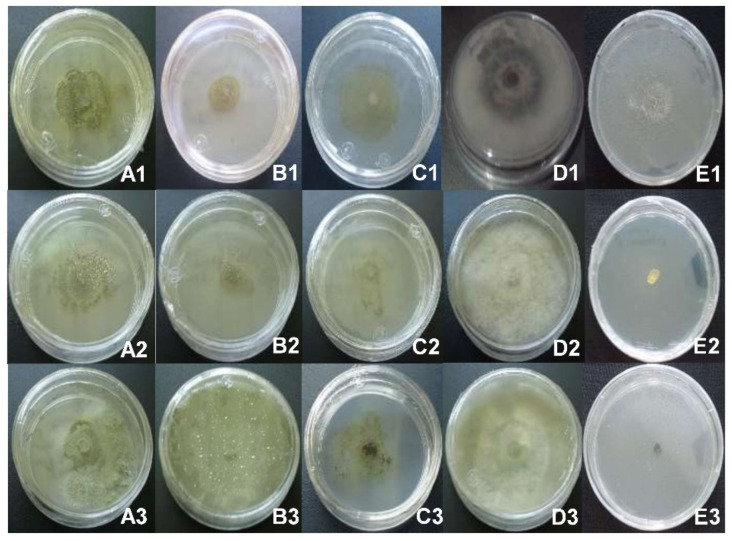
Inhibitory effect of the crude extracts: M5L of *L. hirta* (**A**), M6LFr of *A. ochroleuca* (**B**) and M4LS of *A. porophyllum* (**C**); on the mycelium of *C. gloeosporioides* (A1, B1 and C1), *P. clavispora* (A2, B2 and C2) and *L. pseudotheobromae* (A3, B3 and C3), at a concentration of 5 mg/mL. The negative control was absolute ethanol (**D**), and the positive control Thiabendazole (**E**).

The mycelial growth of blueberry pathogens was inhibited by at least one of the extracts from the plants under study. In the case of the treatments with *L. hirta*, the extract M5L (at a concentration of 5 mg/mL) inhibited 100% of the growth of *P. clavispora* and *C. gloeosporioides*, the same results obtained with the positive controls (Figure 1(A1,A2,E1,E2), Table 1).

The inhibitory activity of this extract against *L. pseudotheobromae* was only 22%, but it was significant (*p* ≤ 0.05) compared to the negative control (Figure 1(A3); Table 1). Other less effective extracts were M8Fl, which inhibited 24% of the growth of *P. clavispora*, and M1L, which inhibited 55% of the growth of *C. gloeosporioides*. A lower concentration of M5L (2 mg/mL) inhibited 21% of the growth of *C. gloeosporioides* (Table 1).

In treatments with *A. ochroleuca*, the most effective extract was M6LFr (at a concentration of 5 mg/mL), completely inhibiting the growth of *P. clavispora* and *C. gloeosporioides*, the same results obtained with positive controls containing Thiabendazole (Figure 1(B1,B2,E1,E2), Table 1). However, it had no effect on the mycelial growth of *L. pseudotheobromae* (Figure 1(B3); Table 1). Furthermore, the M3LFr extract showed significant inhibition (*p* ≤ 0.05) of *P. clavispora* (59%) and *C. gloeosporioides* (48%). The least effective extract was M9R (Table 1).

Regarding the treatments with *A. porophyllum*, the extract with the highest inhibitory activity was M4LS at a concentration of 5 mg/mL, inhibiting 100% of the growth of the three phytopathogens, the same result obtained with positive controls (Figure 1(C1–C3,E1–E3); Table 1). M4LS also showed significant inhibition (*p* ≤ 0.05) of *L. pseudotheobromae* at concentrations of 1 and 2 mg/mL (78.7 and 82.7%, respectively). At a concentration of 5 mg/mL, the M2LS extract inhibited 100% of *C. gloeosporioides*, whereas at 2 mg/mL it inhibited only 18%, with significant differences compared to the negative control. The M7L extract (5 mg/mL) was not very effective against *P. clavispora* and *C. gloeosporioides*. None of the extracts (*L. hirta*, *A. ochroleuca* and *A. porophyllum*) was effective against the mycelium of blueberry fungi at doses of 0.5 mg/mL (Table 1).

### 3.3. Fractions of Crude Extracts and Identification of Bioactive Compounds

The crude extracts that showed 100% antifungal activity in vitro were fractionated and evaluated. Nine of 10 fractions of the M5L extract of *L. hirta* showed significant antifungal activity (*p* < 0.05) compared to the negative control; the F10 fraction showed the highest percentages of inhibition (44.3%) against *C. gloeosporioides*. Few fractions had effect against *P. clavispora*, as was the case of F6, with 11% inhibition (Table 2).

**Figure 2:**
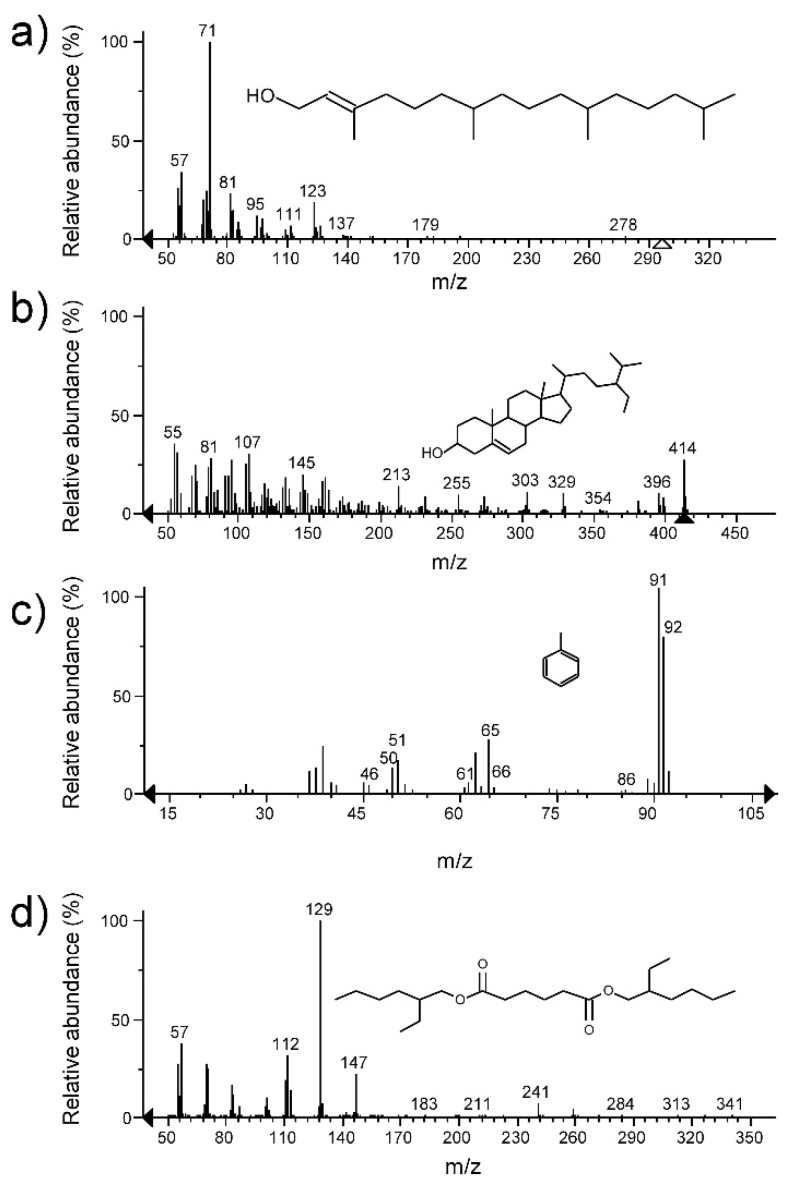
Mass spectra and chemical structures identified in the fractions F6 and F10 from the M5L extract of *L. hirta*, Phytol (**a**) and α-sitosterol (**b**). In the fraction F10 from the M6LFr extract of *A. ochroleuca*, Toluene (**c**). In the fractions F2, F4 and F5 from the M4LS extract of *A. porophyllum*, Hexanedioic acid, bis(2-ethylhexyl) ester (**d**).

The secondary metabolites present in the F6 and F10 fractions of the M5L extract were Phytol (68.09% abundance) for F6 and α-sitosterol (44.80% of abundance) for F10 (Figure 2).

The seventeen fractions from the M6LFr extract of *A. ochroleuca* showed significant antifungal activity (*p* < 0.05) against *P. clavispora* and *C. gloeosporioides* compared to the negative control. Fractions F13 (36%) showed the highest inhibitory activity against *P. clavispora*, while F11 (26.0%) had the highest inhibitory activity against *C. gloeosporioides* (Table 3). The secondary metabolites identified in fractions F13 and F11 from M6LFr extract were Toluene (53.7% abundance) and Benzene, 1,3-bis(3-phenoxyphenoxy)- (100% abundance), respectively (Figure 2).

The M4LS extract of *A. porophyllum* yielded eight fractions with significant antifungal activity (*p* < 0.05) against the three fungi under study. The fractions with the highest percentage of inhibition were F2 (34.9%) against *P. clavispora*, F4 (12.2%) against *C. gloeosporioides* and F5 (16.1%) against *L. pseudotheobromae* (Table 4). Hexanedioic acid, bis (2-ethylhexyl) ester was identified in these three fractions, with percentages of abundance of 84.99%, 91.34% and 100%, respectively (Figure 2).

## 4. Discussion

The diseases affecting blueberry crops in northwestern Michoacán, Mexico, cause significant losses in production. This study identified three fungi that are often isolated from plants with symptoms of dieback and cancers: *P. clavispora*, *C. gloeosporioides* and *L. pseudotheobromae*. The first two have already been reported in blueberry crops by other studies [26,27], but we did not find reports of *L. pseudotheobromae* in blueberry, although it has been reported in other crops [28,29].

Given the significant problems caused by these fungi in the cultivation of blueberry, the use of plant extracts from medicinal plants has been considered as a control alternative [30]. This study shows that extracts of *L. hirta*, *A. ochroleuca* and *A. Porophyllum* have antifungal activity in vitro. Among the best treatments, the inhibitory effect (100%) of the extract M5L (AcOEt) of *L. hirta* on *P. Clavispora* and *C. gloeosporioides* was most prominent. Another study showed that extracts of other species of the same genus (*L. camera*) have an antifungal effect against *F. oxysporum* but not against *C. gloeosporioides* [8]. We found no reports on the antifungal effect of extracts of *L. hirta* against these or other pathogens of blueberry.

Another extract based on *A. ochroleuca* that inhibited 100% of the growth of both pathogens (at a concentration of 5 mg/mL) was M6LFr (AcOEt). These results are important because the antifungal effect of this extract was similar to that of the commercial fungicide Thiabendazole, which was used as positive control. Other studies have noted the antifungal effect of methanol extracts of *A. ochroleuca* against *Fusarium sporotrichum* ATCC 3299, *Aspergillus niger*, *Trichophyton mentagrophytes* and *F. moniliforme* [14].

The M4LS extract (AcOEt) of *A. porophyllum* was the only one that inhibited 100% of the three pathogenic fungi of blueberry, including *L. pseudotheobromae*, which was the most resistant fungus in this study. This is the first report of the antifungal effect of extracts of *A. porophyllum* against phytopathogenic fungi.

To carry out a preliminary approach to the chemical nature of the compounds present in fractions extracts with the highest antifungal activity, a GC-MS analysis was performed. The major compounds in the active fractions of the M5L extract of *L. hirta* were the diterpene Phytol and the triterpene α-Sitosterol. Phytol is a common compound in extracts of plants such as *Porophyllum linaria* [31], *Vitex negundo* [32], *Rhaponticum acaule* [33] and *Apium nodiflorum* [34]. It has also been reported as one of the main components of the essential oil of *A. nodiflorum*, which showed antifungal activity against yeasts, dermatophytes, and *Aspergillus* spp. [34]. The triterpene α-sitosterol has been isolated from *Leucas aspera* [35].

The compound identified in the most active fraction of the M6LFr extract of *A. ochroleuca* was Benzene, 1,3-bis (3-phenoxyphenoxy)-. The major compound in essential oil of *Bridelia micrantha*, Benzene, 1,3-bis (3-phenoxyphenoxy)- has shown antimicrobial activity against *Mycobacterium tuberculosis* [36]. However, there are no reports of this compound having antifungal activity against *P. clavispora* and *C. gloeosporioides*.

A single compound was identified in the three most active fractions from the M4LS extract of *A. porophyllum*: Hexanedioic acid, bis(2-ethylhexyl) ester. This compound has been isolated from the roots of *Stellera chamaejasme* [37], the stem of *Hugonia mystax* [38], and even from *Streptomyces* sp. [39]. This compound has shown antifungal activity against *Monilinia fructicola* [37] and *Fusarium* sp. [39]. However, it is necessary to carry out experiments that lead us to compounds purification and confirm their antifungal activity.

## 5. Conclusions

This study shows that extracts of the medicinal plants *L. hirta*, *A. ochroleuca* and *A. porophyllum* inhibited 100% of the mycelial growth in vitro of *P. clavispora*, *C. gloeosporioides* and *L. pseudotheobromae* at a concentration of 5 mg/mL. Analysis by GC-MS indicates that the bioactive compounds present in the most effective extracts are the diterpene Phytol; the triterpenes α-Sitosterol; and Toluene, Benzene, 1,3-bis(3-phenoxyphenoxy)- and Hexanedioic acid, bis(2-ethylhexyl) ester. This demonstrates the potential of *L. hirta*, *A. ochroleuca* and *A. porophyllum* as sources of antifungal compounds.

## Figures and Tables

**Table 1:** Percentage of mycelial growth inhibition of phytopathogenic fungi of blueberry by crude extracts of *L. hirta*, *A. ochroleuca* and *A. porophyllum*.

Extracts ^Ɛ^		Concentration (mg/mL)	Inhibition (%)		
			*P. clavispora*	*C. gloeosporioides*	*L. pseudotheobromae*
	M1L (EtOH)	0.5, 1, 2	0 ^g^	0 ^j^	0 ^e^
		5	5.4 ^e^	55 ^b^	0 ^e^
	M5L (AcOEt)	0.5, 1	0 ^g^	0 ^j^	0 ^e^
		2	0 ^g^	21 ^e^	0 ^e^
*L. hirta*		5	100 ^a^	100 ^a^	22 ^d^
	M8Fl (AcOEt)	0.5, 1, 2	0 ^g^	0 ^j^	0 ^e^
		5	24 ^c^	30 ^d^	0 ^e^
	M11Fl (EtOH)	0.5, 1, 2, 5	0 ^g^	0 ^j^	0 ^e^
	M3LFr (EtOH)	0.5, 1, 2	0 ^g^	0 ^j^	0 ^e^
		5	59 ^b^	48 ^c^	0 ^e^
	M6LFr (AcOEt)	0.5, 1, 2	0 ^g^	0 ^j^	0 ^e^
*A. ochroleuca*		5	100 ^a^	100 ^a^	0 ^e^
	M9R (AcOEt)	0.5, 1, 2	0 ^g^	0 ^j^	0 ^e^
		5	4 ^f^	5 ^i^	0 ^e^
	M12R (EtOH)	0.5, 1, 2, 5	0 ^g^	0 ^j^	0 ^e^
	M2LS (EtOH)	0.5, 1	0 ^g^	0 ^j^	0 ^e^
		2	0 ^g^	18 ^f^	0 ^e^
		5	0 ^g^	100 ^a^	0 ^e^
	M4LS (AcOEt)	0.5	0 ^g^	0 ^j^	0 ^e^
*A. porophyllum*		1	0 ^g^	0 ^j^	79 ^c^
		2	0 ^g^	13 ^g^	83 ^b^
		5	100 ^a^	100 ^a^	100 ^a^
	M7L (AcOEt)	0.5, 1, 2	0 ^g^	0 ^j^	0 ^e^
		5	7 ^d^	10 ^h^	0 ^e^
	M10L (EtOH)	0.5, 1, 2, 5	0 ^g^	0 ^j^	0 ^e^
Absolute control *		-	0 ^g^	0 ^j^	0 ^e^
Negative control		-	0 ^g^	0 ^j^	0 ^e^
Positive control		5	100 ^a^	100 ^a^	100 ^a^

Different letters in the same column indicate significant differences (*p* ≤ 0.05). ^Ɛ^ M1-M12 = leaf extracts (L) and flower extracts (Fl) of *L. hirta*; leaf-fruit extracts (LFr) and root extracts (R) of *A. ochroleuca*; leaf-stem extracts (LS) and leaf extracts (L) of *A. porophyllum*. Absolute ethanol (EtOH) and ethyl acetate (AcOEt) were used as solvents. * Absolute control (PDA + fungus). Negative control (PDA + fungus + EtOH). Positive control (PDA + fungus + Thiabendazole, TECTO 60^®^).

**Table 2:** Percentage of mycelial growth inhibition of *P. clavispora* and *C. gloeosporioides* by fractions from the M5L extract of *L. hirta*.

Fractions	Concentration (mg/mL)	Inhibition (%)	
		*P. clavispora*	*C. gloeosporioides*
F1	1.8	0 ^e^	8.2 ^g^
F2	1.8	0 ^e^	12.9 ^e^
F3	1.8	0 ^e^	15.3 ^d^
F4	2.4	0 ^e^	0 ^j^
F5	1.8	0 ^e^	20.0 ^c^
F6	2.4	11.0 ^b^	9.4 ^f^
F7	1.8	0 ^e^	6.3 ^h^
F8	1.2	10.6 ^c^	0 ^j^
F9	2.4	10.6 ^c^	4.3 ^i^
F10	0.6	2.0 ^d^	44.3 ^b^
Absolute control *	-	0 ^e^	0 ^j^
Negative control	-	0 ^e^	0 ^j^
Positive control	5	100 ^a^	100 ^a^

Different letters in the same column indicate significant differences (*p* ≤ 0.05). * Absolute control (PDA + fungus). Negative control (PDA + fungus + EtOH). Positive control (PDA + fungus + Thiabendazole, TECTO 60^®^).

**Table 3:** Percentage of mycelial growth inhibition of *P. clavispora* and *C. gloeosporioides* by fractions from the M6LFr extract of *A. ochroleuca*.

Fractions	Concentration (mg/mL)	Inhibition (%)	
		*P. clavispora*	*C. gloeosporioides*
F1	1.5	8.7 ^j^	4.0 ^n^
F2	3.6	8.7 ^j^	18 ^g^
F3	3	18.7 ^i^	6.7 ^m^
F4	2.4	18.7 ^i^	12.0 ^j^
F5	4.8	1.3 ^l^	8.0 ^l^
F6	2.4	22.7 ^g^	20.7 ^e^
F7	3	1.3 ^l^	24.0 ^c^
F8	1.8	28.0 ^e^	20.0 ^f^
F9	1.2	28.0 ^e^	21.3 ^d^
F10	0.6	34.7 ^c^	20.0 ^f^
F11	1.8	27.3 ^e^	26.0 ^b^
F12	1.2	34.7 ^c^	18.0 ^g^
F13	3	36.0 ^b^	16.0 ^h^
F14	1.2	24.0 ^f^	22.0 ^d^
F15	1.2	20.7 ^h^	13.3 ^i^
F16	1.2	4.0 ^k^	10.0 ^k^
F17	1.2	33.3 ^d^	20.0 ^f^
Absolute control *	-	0 ^m^	0 ^o^
Negative control	-	0 ^m^	0 ^o^
Positive control	5	100 ^a^	100 ^a^

Different letters in the same column indicate significant differences (*p* ≤ 0.05). * Absolute control (PDA + fungus). Negative control (PDA + fungus + EtOH). Positive control (PDA + fungus + Thiabendazole, TECTO 60^®^).

**Table 4:** Percentage of mycelial growth inhibition of *P. clavispora*, *C. gloeosporioides* and *L. pseudotheobromae* by fractions from the M4LS crude extract of *A. porophyllum*.

Fractions	Concentration (mg/mL)	Inhibition (%)		
		*P. clavispora*	*C. gloeosporioides*	*L. pseudotheobromae*
F1	6.6	29.8 ^c^	7.8 ^d^	4.7 ^d^
F2	1.8	34.9 ^b^	6.7 ^e^	14.1 ^c^
F3	3	14.9 ^e^	7.5 ^d^	14.1 ^c^
F4	3	26.3 ^d^	12.2 ^b^	1.6 ^g^
F5	3	11.0 ^f^	5.9 ^f^	16.1 ^b^
F6	1.2	6.7 ^g^	7.5 ^d^	3.1 ^e^
F7	2.4	6.7 ^g^	8.6 ^e^	2.8 ^f^
F8	4.2	5.9 ^h^	6.7 ^e^	0 ^h^
Absolute control *	-	0 ^i^	0 ^g^	0 ^h^
Negative control	-	0 ^i^	0 ^g^	0 ^h^
Positive control	5	100 ^a^	100 ^a^	100 ^a^

Different letters in the same column indicate significant differences (*p* ≤ 0.05). * Absolute control (PDA + fungus). Negative control (PDA + fungus + EtOH). Positive control (PDA + fungus + Thiabendazole, TECTO 60^®^).

## Data Availability

The authors declare that the data supporting the findings of this study are available within the article.

## References

[1] SIAP-SAGARPA Servicio de Información Agroalimentaria y Pesquera-Secretaría de Agricultura, Ganadería, Desarrollo Rural, Pesca y Alimentación. https://nube.siap.gob.mx/cierreagricola/.

[2] Bristow P., Ramsdell D., Caruso F., Ramsdell D. (1995). Botrytis blight. Compendium of Blueberry and Cranberry Diseases.

[3] Wharton P.S., Schilder A.C. (2008). Novel infection strategies of Colletotrichum acutatum on ripe blueberry fruit. Plant Pathol..

[4] Espinoza J.G., Briceño E.X., Keith L.M., Latorre B.A. (2008). Canker and twig dieback of blueberry caused by *Pestalotiopsis* spp. and a *Truncatella* sp. in Chile. Plant Dis..

[5] Sammonds J., Billones R., Rocchetti M., Ridgway H.J., Walter M., Jaspers M.V. (2009). Survey of blueberry farms for Botryosphaeria dieback and crown rot pathogens. N. Z. Plant Prot..

[6] Kong C.S., Qiu X.L., Yi K.S., Yu X.F., Yu L. (2010). First Report of Neofusicoccum vitifusiforme Causing Blueberry Blight of Blueberry in China. Plant Dis..

[7] Díaz P., Cabrera A., Alem D., Larrañaga P., Ferreira F., Rizza M.D. (2011). Antifungal activity of medicinal plant extracts against phytopathogenic fungus *Alternaria* spp.. Chil. J. Agric. Res..

[8] Mdee L.K., Masoko P., Eloff J.N. (2009). The activity of extracts of seven common invasive plant species on fungal phytopathogens. S. Afr. J. Bot..

[9] Salgado-Garciglia R., Molina-Torres J., López-Meza J.E., Loeza-Lara P.D. (2008). Efecto del extracto crudo y los compuestos bioactivos de Heliopsis longipes sobre la incidencia de la antracnosis, micorrización y nodulación del frijol. Agrociencia.

[10] Ribera A.E., Zuñiga G. (2012). Induced plant secondary metabolites for phytopatogenic fungi control: A review. J. Soil Sci. Plant Nutr..

[11] Madariaga-Mazón A., Hernández-Alvarado R.B., Noriega-Colima K.O., Osnaya-Hernández A., Martinez-Mayorga K. (2019). Toxicity of secondary metabolites. Phys. Sci. Rev..

[12] Montes-Belmont R., Cruz-Cruz V., Martínez-Martínez G., Sandoval-García G., García-Licona R., Zilch-Domínguez S., Bravo-Luna L., Bermúdez-Torres K., Flores-Moctezuma H.E., Carvajal-Moreno M. (2000). Propiedades antifúngicas en plantas superiores. Análisis retrospectivo de investigaciones. Rev. Mex. Fitopatol..

[13] López-Villafranco M.E., Aguilar-Contreras A., Aguilar-Rodríguez S., Xolalpa-Molina S. (2017). Las Verbenaceae empleadas como recurso herbolario en México: Una revisión etnobotánica-médica The Verbenaceae used as an herbal resource in Mexico: An ethnobotanical-medical review. Polibotánica.

[14] Reyes F.D., Peña C.J., Canales M., Jiménez M., Meráz S., Hernandez T. (2011). Antimicrobial activity of Argemone ochroleuca Sweet (Chicalote). Boletín Latinoam. Caribe Plantas Med. Aromáticas.

[15] Villareal J.Á., Villaseñor J.L., Sosa V. (2005). Flora de Veracruz. Compositae. Tribu Tageteae.

[16] Agrios G. (2005). Plant Pathology.

[17] Raeder U., Broda P. (1985). Rapid preparation of DNA from filamentous fungi. Lett. Appl. Microbiol..

[18] Barrera-Necha L.L., Bautista-Baños S. (2008). Actividad Antifúngica de Polvos, Extractos y Fracciones de Cestrum nocturnum L. Sobre el Crecimiento Micelial de Rhizopus stolonifer (Ehrenb.:Fr.) Vuill. Rev. Mex. Fitopatol..

[19] Castillo F., Hernández D., Gallegos G., Mendez M., Rodríguez R., Reyes A., Aguilar C.N. (2010). In vitro antifungal activity of plant extracts obtained with alternative organic solvents against Rhizoctonia solani Kühn. Ind. Crops Prod..

[20] Barrera-Figueroa B.E., Loeza-Lara P.D., Hernández-García A., López-Mesa J.E., Molina-Torres J., del Río-Torres R.E.N., Martínez-Pacheco M.M., López-Gómez R., Salgado-Garciglia R. (2011). Antibacterial activity of flower extracts from *Helenium mexicanum* H.B.K. Emir. J. Food Agric..

[21] Waksmundzka-Hajnos M., Sherma J., Kowalska T., Waksmundzka-Hajnos M., Sherma J., Kowalska T. (2008). Overview of the Field of TLC in Phytochemistry and the Structure of the Book. Thin Layer Chromatography in Phytochemistry.

[22] Sánchez-Pérez J.D.L., Jaimes-Lara M.G., Salgado-Garciglia R., López-Meza J.E. (2009). Root extracts from Mexican avocado (*Persea americana* var. drymifolia) inhibit the mycelial growth of the oomycete Phytophthora cinnamomi. Eur. J. Plant Pathol..

[23] Pandey D.K., Tripathi N.N., Tripathi R.D., Dixit S.N. (1982). Fungitoxic and phytotoxic properties of the essential oil of *Hyptis suaveolens*. J. Plant Dis. Prot..

[24] Adams R.P. (2007). Identification of Essential Oil Components by Gas Chromatography/Mass Spectrometry.

[25] Ruiz-Sánchez E., Mejía-Bautista M.A., Cristóbal-Alejo J., Valencia-Botín A., Reyes-Ramírez A. (2014). Actividad antagónica de filtrados de Bacillus subtilis contra Colletotrichum gloeosporioides (Penz.). Rev. Mex. Cienc. Agrícolas.

[26] Mondragón F.A., López M.J., Ochoa A.S., Gutiérrez C.M. (2012). Hongos Asociados a la Parte Aérea del Arándano en Los Reyes, Michoacán, México. Rev. Mex. Fitopatol..

[27] Sabaratnam S. (2018). Blueberry Anthracnose (Ripe Rot).

[28] Tovar-Pedraza J.M., Mora-Aguilera J.A., Nava-Díaz C., Téliz-Ortiz D., Valdovinos-Ponce G., Villegas-Monter Á., Hernández-Morales J. (2012). Identification, pathogenicity, and histopathology of lasiodiplodia theobromae on mamey sapote grafts In Guerrero, México. Agrociencia.

[29] Sandoval-Sánchez M., Nieto-Ángel D., Sandoval-Islas J.S., Téliz-Ortiz D., Orozco-Santos M., Silva-Rojas H.V. (2013). Hongos asociados a pudrición del pedúnculo y muerte descendente del mango (*Mangifera Indica* L.). Agrociencia.

[30] Kalidindi N., Thimmaiah N.V., Jagadeesh N.V., Nandeep R., Swetha S., Kalidindi B. (2015). Antifungal and antioxidant activities of organic and aqueous extracts of *Annona squamosa* Linn. leaves. J. Food Drug Anal..

[31] Juárez Z.N., Hernández L.R., Bach H., Sánchez-Arreola E., Bach H. (2015). Antifungal activity of essential oils extracted from *Agastache mexicana* ssp. xolocotziana and *Porophyllum linaria* against post-harvest pathogens. Ind. Crops Prod..

[32] Praveen Kumar P., Kumaravel S., Lalitha C. (2010). Screening of antioxidant activity, total phenolics and GC-MS study of Vitex negundo. African J. Biochem. Res..

[33] Boussaada O., Ammar S., Saidana D., Chriaa J., Chraif I., Daami M., Helal A.N., Mighri Z. (2008). Chemical composition and antimicrobial activity of volatile components from capitula and aerial parts of Rhaponticum acaule DC growing wild in Tunisia. Microbiol. Res..

[34] Maxia A., Falconieri D., Piras A., Porcedda S., Marongiu B., Frau M.A., Gonçalves M.J., Cabral C., Cavaleiro C., Salgueiro L. (2012). Chemical Composition and Antifungal Activity of Essential Oils and Supercritical CO 2 Extracts of *Apium nodiflorum* (L.) Lag. Mycopathologia.

[35] Prajapati M., Patel J., Modi K., Shah M. (2010). Leucas aspera: A review. Pharmacogn. Rev..

[36] Green E., Obi L.C., Samie A., Bessong P.O., Ndip R.N. (2011). Characterization of n-Hexane sub-fraction of Bridelia micrantha (Berth) and its antimycobacterium activity. BMC Complement. Altern. Med..

[37] Xue-Na B., Cheng J., Liang W., Lan-Qing M., Yu-Bo L., Guang-Lu S., You-Nian W., Zhu E., Sambath S. (2012). Antifungal activity of extracts by supercritical carbon dioxide extraction from roots of *Stellera chamaejasme* L. and analysis of their constituents using GC-MS. Advances in Intelligent and Soft Computing.

[38] Vimalavady A., Kadavul K. (2013). Phytocomponents identified on the various extracts of stem of *Hugonia mystax* L. (Linaceae). Pelagia Res. Libr. Eur. J. Exp. Biol..

[39] Elleuch L., Shaaban M., Smaoui S., Mellouli L., Karray-Rebai I., Fourati-Ben Fguira L., Shaaban K.A., Laatsch H. (2010). Bioactive secondary metabolites from a new terrestrial streptomyces sp. TN262. Appl. Biochem. Biotechnol..

